# Radiation Fibrosis After Stereotactic Body Radiation Therapy for Osseous Metastases: A Case Report

**DOI:** 10.7759/cureus.28925

**Published:** 2022-09-08

**Authors:** Michael R Kessler, Austin P Dove, Austin N Kirschner

**Affiliations:** 1 Medicine, University of Tennessee Health Science, Memphis, USA; 2 Radiation Oncology, Vanderbilt University Medical Center, Nashville, USA

**Keywords:** stereotactic body radiation therapy, oligometastatic, renal cell carcinoma, myositis, radiation-induced fibrosis

## Abstract

Radiation-induced fibrosis is a potentially severe late complication after high-dose radiotherapy. Over the last decade, there has been increasing use of stereotactic body radiation therapy (SBRT) to treat both primary and metastatic malignancies. While there has been evolving evidence of appropriate dose constraints for certain organs receiving hypofractionated radiotherapy, the risk, and appropriate dose constraints to limit the risk of radiation-induced muscle fibrosis are poorly defined. In this report, two patients are presented who underwent SBRT for osseous oligometastatic renal cell carcinoma. While the treatment was well-tolerated with no acute toxicities and complete local control of the metastasis, both patients experienced late toxicity of radiation-induced fibrosis in the adjacent musculature. In both cases, toxicity was nonresponsive to medical interventions and was severe enough to require surgical resection of the affected tissue. Following surgery, both patients reported improved pain relief and mobility. Further studies are needed to explore the dose constraints that may reduce the risk of radiation-induced muscle fibrosis in five-fraction treatment.

## Introduction

While radiotherapy is an effective cancer treatment, the effects of radiation can impact both malignant cells and the normal surrounding tissues in the treatment field. The late toxicities of radiation-induced damage to normal tissues can be seen months to years following treatment. Late toxicity is thought to develop as a function of total radiation dose, dose per treatment, volume of tissue treated, and number of fractions [1]. One example of late tissue toxicity is radiation-induced fibrosis (RIF), which is characterized by an abnormal wound healing response that leads to excessive deposition of collagen at the radiation treatment site [2]. Radiation fibrosis syndrome describes the clinical sequelae that result from RIF, which can involve the neuromuscular, musculoskeletal, integumentary, and visceral systems. In particular, skeletal muscle involvement causes muscle weakness, atrophy, pain, and decreased mobility, which is important considerations when planning radiation treatment, particularly in the palliative setting [3].

Historically, the risk of RIF was shown to be significantly higher in a single fraction with total doses between 10-20 Gy [4]. With the increasing use of hypofractionated treatments with stereotactic body radiation therapy (SBRT), appropriate dose constraints for RIF should be re-examined. SBRT provides a high biological dose to the tumor with steep dose gradients to minimize the dose to surrounding critical structures, which has been particularly useful in treatments near sensitive structures, such as spinal metastases [5]. While potentially limiting dose to critical structures, SBRT is not without inherent risks due to its large dose per fraction. Despite higher rates of local control compared to conventional external beam radiotherapy, SBRT has been shown to have an increased risk of certain toxicities, such as vertebral compression fractures, likely due to higher biologically effective doses (BED) [6]. Lockney et al. have recently characterized an underreported late side effect of radiation-induced myositis, however, there is limited literature exploring the late RIF complications of SBRT [7]. With increasing utilization of SBRT especially in radioresistant tumor histologies, such as renal cell carcinoma, strategies to mitigate the risk of late toxicities such as RIF should be investigated. Herein, we report two cases of RIF and discuss the current therapeutic management of RIF including physical therapy, pharmaceuticals, and interventional treatments [8].

## Case presentation

Case 1

In August 2017, a 38-year-old man was initially diagnosed with renal cell carcinoma after presenting to the ED with gross hematuria. After staging imaging showed no evidence of metastatic disease, he underwent a right radical nephrectomy revealing a pathologic T2aN0MX clear cell renal cell carcinoma with rhabdoid features with negative margins. He was initiated on adjuvant immunotherapy from October 2017 to March 2018. Unfortunately, immunotherapy was discontinued following metastatic progression in the lungs. In October 2020, he was found to have a new lytic lesion in the right scapula measuring 3.8 x 7.3 x 5.3 cm with evidence of muscle invasion. He underwent SBRT to the right scapular lesion with a dose of 40 Gy in five fractions in November 2020.

**Figure 1:**
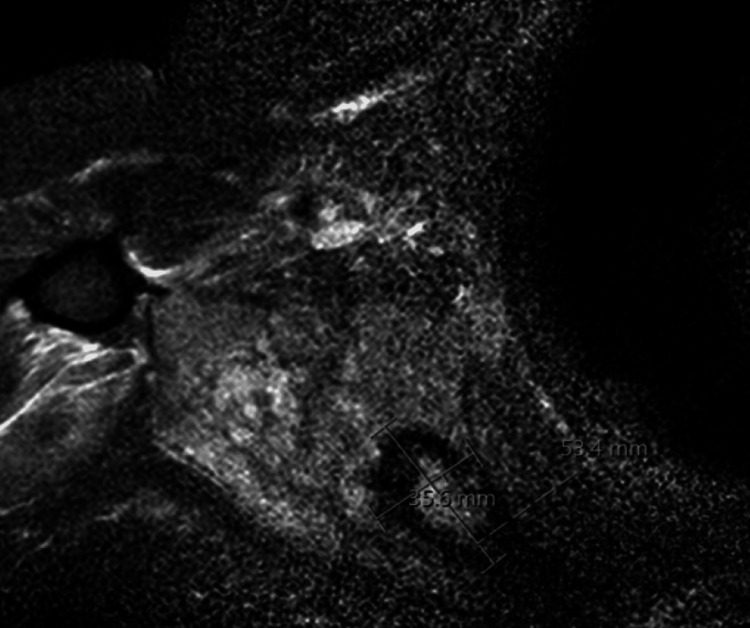
Post-treatment MRI Post-treatment T2 axial MRI showing evidence of edema and enhancement in previously treated region

Shortly after radiotherapy, he reported improvements in shoulder pain. However, about three months later, his pain returned and progressively worsened. He was initially managed over the next several months with physical therapy, ibuprofen, muscle relaxers, steroids, and opiates. During pain flare up, CT imaging showed no evidence of disease progression or fractures. Despite this management, he continued to have severe discomfort. In April 2021, he underwent a surveillance MRI that showed enhancement and edema in the peri-scapular musculature (Figure 1). A PET/CT scan was not obtained. Orthopedic oncology was consulted and performed a radical resection of the inferior right scapula and associated inflamed tissue with pathology showing evidence of inflammation and fibrosis consistent with RIF, and no residual tumor. Following surgery, he reported significantly improved pain and increased shoulder range of motion. 

Case 2

In 2016, a 54-year-old woman presented after an incidental renal mass was found on imaging with no evidence of metastatic disease. She underwent right radical nephrectomy revealing a pathologic T3aN0MX renal cell carcinoma with negative margins. Unfortunately, several months following surgery, she was found to have distant metastatic disease with biopsy-proven mediastinal adenopathy. She was subsequently started on systemic therapy with a combination of axitinib and avelumab. She initially had a good response to systemic therapy with no evidence of progression for about three years. In 2019, she reported progressively worsening left hip pain with CT imaging showing soft tissue density in the left anterior superior iliac spine measuring 3.4 x 1.8 cm with associated lytic and sclerotic bone changes consistent with metastatic disease. She underwent SBRT to the left hip lesion with a dose of 40 Gy in five fractions in April 2019.

Following radiotherapy, she initially experienced pain relief with no evidence of progression on three-month-interval surveillance imaging. However, in December 2019, she began having progressively worsening hip pain. Over the next few months, the pain was managed with physical therapy, ibuprofen, and opiates. Despite conservative management, the pain was persistent and limited her range of motion. In June 2021, imaging revealed a fracture at the previously treated left iliac crest. She was referred to orthopedic oncology with recommendation for type 1 hemipelvectomy. In July 2021, she underwent surgical resection with pathology showing evidence of radiation fibrosis, tissue necrosis, and no recurrent tumor.

## Discussion

Multiple prospective trials have reported dosimetric constraints to critical tissues for SBRT to the spine, such as the spinal cord, cauda equina, lumbosacral plexus, and visceral organs [9]. A subsequent review highlighted the available literature regarding dose limits and mitigation strategies for radiation myelopathy, plexopathy, and vertebral compression fracture after spine SBRT [10]. Despite this literature on SBRT toxicity, cases of SBRT-associated myositis appear limited [11]. One retrospective cohort from Lockney et al. has detailed the rare late complication of radiation myositis after SBRT to the spine [7]. In this study, the authors identified 11 patients who had developed this manifestation at a rate of 1.6% of patients receiving spinal SBRT. The median time to the development of RIF was approximately four months after SBRT. Interestingly, the study concluded that a single fraction SBRT could be associated with a higher risk of radiation myositis. While the incidence of cases in literature appears sparse, there is likely an under-reported rate of RIF in the modern era of radiation oncology with increased use of SBRT and the ability for dose escalation with improved technology. Additionally, immune checkpoint inhibitors have been associated with myositis toxicity [12]. In an era of increased synergistic use of SBRT and immunotherapy, the potential increased risk of myositis has not been well described. 

**Figure 2:**
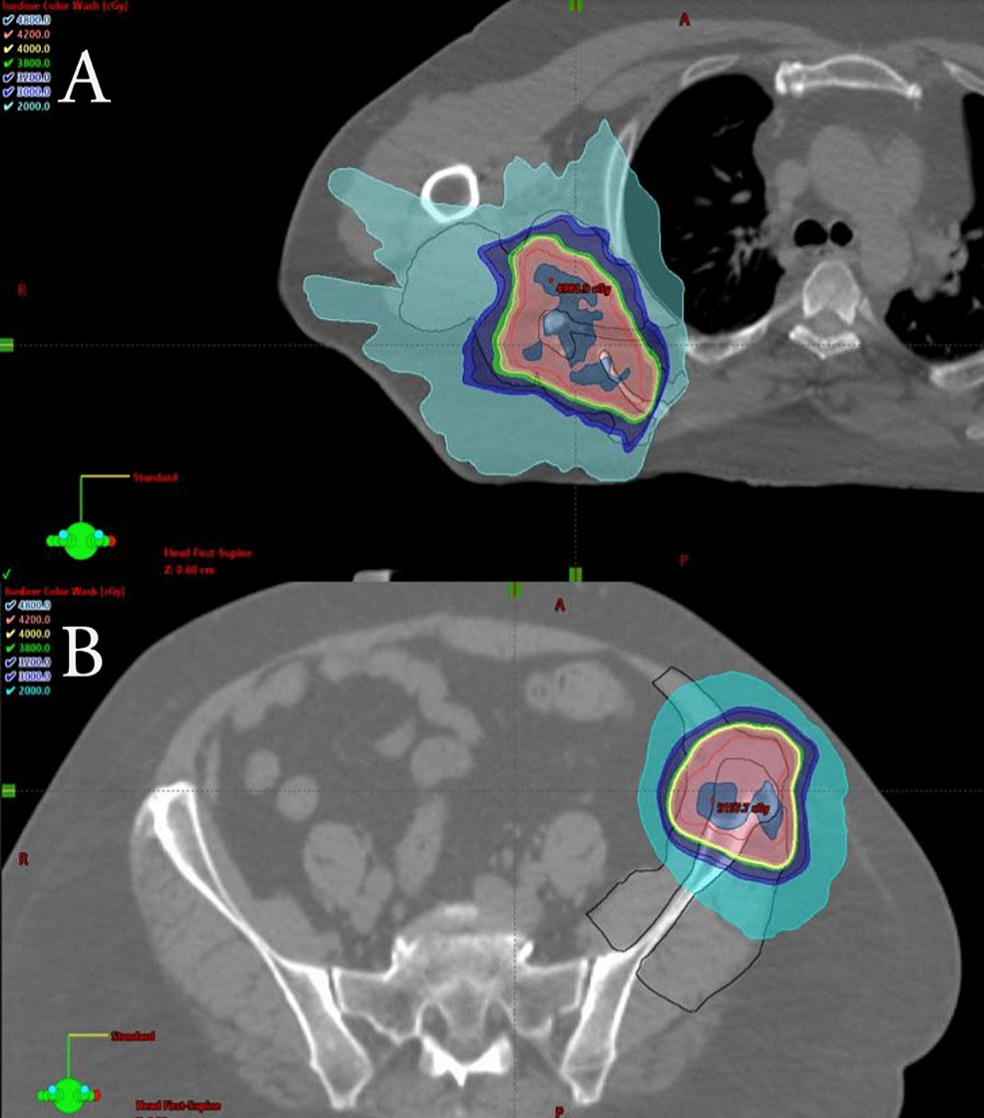
Stereotactic Body Radiation Therapy (SBRT) Treatment Plans Outlined are images of isodose color wash for each patient’s body site (scapula (A), Iliac (B)). Muscle contour is outlined in black. Prescription dose (40Gy) is outlined in yellow.

In our study, we reviewed two non-spine SBRT cases of RIF using five fraction SBRT. In both cases, patients required surgical intervention for pain relief. After extensive pathologic review in each case, residual cancer was not identified with pathological findings consistent with radiation-induced fibrosis characterized by excessive chronic inflammation, wound healing, and collagen deposition [2]. When analyzing our two cases of RIF, we retrospectively contoured all skeletal muscle delineated within 5 cm of the target PTV as outlined in Figure 2.

Dose-volume histogram data, as outlined in Table 1, was used to determine the total volume (cc) of muscle that received at least 20 Gy in five fractions for each case. These two patients had at least 50 cc of muscle receiving at least 40 Gy. Using the α/β ratio of 5.0 for renal cell carcinoma, 40 Gy in five fractions is equivalent to a BED of 104 Gy with an EQD2 of 74.29 Gy. In Lockney et al, the median BED was 82.7 Gy, and the median equivalent dose (EQD2) was 55.1 Gy. While they describe median BED to the post-treatment myositis-involved muscle, our report analyzes the pre-treatment muscle volume receiving high doses of radiation, which may correlate with the risk of RIF and help inform an algorithm for reducing risk in the radiation treatment planning process. We hypothesize that limiting the total volume of muscle receiving the full prescription dose could limit the risk of RIF and myositis.

**Table 1:** Muscle Dose Volumes Muscle tissue volumes for two patients experiencing severe stereotactic body radiation therapy (SBRT)-induced fibrosis requiring surgical resection for management. Muscle is contoured on the CT simulation planning scan, and the volume of muscle receiving each radiation dose level is determined. The target prescription dose of 4000 cGy is in bold, suggesting a cutoff below which may reduce the risk of severe radiation-induced fibrosis.

5-Fraction SBRT Dose (cGy)	Case 1 Muscle Volume (cm^3^)	Case 2 Muscle Volume (cm^3^)
5200	0.0	0.0
4800	74.8	6.6
4400	201.3	35.8
4000	286.2	52.9
3600	356.1	63.2
3200	428.4	73.0
2800	520.5	85.2
2400	641.4	102.6
2000	739.6	128.4

Restricting the risk of RIF is critical since current management is limited in its ability to slow or reverse the fibrotic process. Initial management of RIF involves physical therapy and exercise regimens meant to loosen the involved tissues and reduce tissue scarring. Current early evidence supports the developing idea that physiotherapy can be beneficial to post-radiotherapy quality of life, which was studied in mostly breast cancer patients [13]. Given the variety of normal tissues exposed to radiation, radiation fibrosis presentation can vary significantly, requiring referral to cancer rehabilitation specialists for individualized evaluation and treatment, which tends to focus on expanding fibrotic tissues and strengthening atrophic muscles [14]. Medical management of RIF should include NSAIDs for the analgesic and anti-inflammatory properties, and insufficient response can be treated by corticosteroids for active myositis. Alternative pharmacologic approaches to RIF include a combination of pentoxifylline and vitamin E, mostly studied in the setting of head and neck irradiation and osteoradionecrosis of the jaw [15] and breast radiation [8]. Furthermore, there is evidence to suggest these agents can be employed as maintenance therapy after radiation to prevent the insidious development of fibrosis [16]. Of note, outcome measures for RIF exhibit wide variability given the largely subjective nature of pain, range of motion, and quality of life. Jacobson et al. did not identify a significant difference in the above subjective measures and reported a significant difference in a standardized measurement of tissue compliance [16]. Botulinum toxin injections have shown symptomatic improvement in a myriad of late radiation fibrosis complications, including cervical dystonia, painful muscle spasms, trismus, and spasticity [17]. In addition, targeted therapies for the molecular pathways of tissue fibrosis and allogeneic bone marrow-derived stem cells have been studied in pre-clinical models, showing promise in the treatment of RIF [18,19]. Further research will be necessary to advance the pharmacologic management of RIF.

Limitations of this case report include the small sample size and lack of controlled methodology that are present in this type of analysis. Outcome conclusions cannot be drawn without a proper control group, and larger, controlled retrospective and prospective studies are required to reliably identify the relationship between radiation dose and late muscle fibrosis. Our case report hypothesizes a clinically reasonable preliminary dose constraint for further investigation. Most notably, further dosimetric analysis may offer the ability to stratify patients at higher risk of SBRT-associated fibrosis. This higher-risk group may benefit from prophylactic and/or maintenance anti-inflammatory pharmacologic therapy to prevent and manage the insidious onset of fibrosis.

## Conclusions

In summary, this report indicates that radiation-induced fibrosis of the muscle may become severe enough to require surgical resection of the inflamed tissue several months after high-dose stereotactic body radiotherapy. We recommend the initiation of physical therapy and anti-inflammatory regimens if symptoms and imaging suggests no disease progression and concern for RIF. Operative management provides symptomatic improvement in severe cases and referral to surgery may benefit those with refractory pain and decreased range of motion.
